# CT-Based Radiomics Score Can Accurately Predict Esophageal Variceal Rebleeding in Cirrhotic Patients

**DOI:** 10.3389/fmed.2021.745931

**Published:** 2021-11-04

**Authors:** Dongxiao Meng, Yingnan Wei, Xiao Feng, Bing Kang, Ximing Wang, Jianni Qi, Xinya Zhao, Qiang Zhu

**Affiliations:** ^1^Department of Gastroenterology, Shandong Provincial Hospital, Cheeloo College of Medicine, Shandong University, Jinan, China; ^2^Department of Gastroenterology, Shandong Provincial Hospital Affiliated to Shandong First Medical University, Jinan, China; ^3^Department of Radiology, Shandong Provincial Hospital, Cheeloo College of Medicine, Shandong University, Jinan, China; ^4^Department of Radiology, Shandong Provincial Hospital Affiliated to Shandong First Medical University, Jinan, China; ^5^Department of Central Laboratory, Shandong Provincial Hospital Affiliated to Shandong University, Jinan, China

**Keywords:** portal hypertension, non-invasive, computed tomography, radiomics, esophageal variceal rebleeding

## Abstract

**Purpose:** This study aimed to develop a radiomics score (Rad-score) extracted from liver and spleen CT images in cirrhotic patients to predict the probability of esophageal variceal rebleeding.

**Methods:** In total, 173 cirrhotic patients were enrolled in this retrospective study. A total of 2,264 radiomics features of the liver and spleen were extracted from CT images. Least absolute shrinkage and selection operator (LASSO) Cox regression was used to select features and generate the Rad-score. Then, the Rad-score was evaluated by the concordance index (C-index), calibration curves, and decision curve analysis (DCA). Kaplan–Meier analysis was used to assess the risk stratification ability of the Rad-score.

**Results:** Rad-score_Liver_, Rad-score_Spleen_, and Rad-score_Liver−Spleen_ were independent risk factors for EV rebleeding. The Rad-score_Liver−Spleen_, which consisted of ten features, showed good discriminative performance, with C-indexes of 0.853 [95% confidence interval (CI), 0.776–0.904] and 0.822 (95% CI, 0.749–0.875) in the training and validation cohorts, respectively. The calibration curve showed that the predicted probability of rebleeding was very close to the actual probability. DCA verified the usefulness of the Rad-score_Liver−Spleen_ in clinical practice. The Rad-score_Liver−Spleen_ showed good performance in stratifying patients into high-, intermediate- and low-risk groups in both the training and validation cohorts. The C-index of the Rad-score_Liver−Spleen_ in the hepatitis B virus (HBV) cohort was higher than that in the non-HBV cohort.

**Conclusion:** The radiomics score extracted from liver and spleen CT images can predict the risk of esophageal variceal rebleeding and stratify cirrhotic patients accordingly.

## Introduction

Esophageal variceal (EV) bleeding is one of the most serious complications in cirrhotic patients with portal hypertension (1). Although several recommended treatments are applied, patients who recover from the first episode of EV bleeding have a high risk of 1-year rebleeding (approximately 60%), with a mortality rate of up to 33% (2). EV rebleeding may lead to a series of complications, such as hepatic encephalopathy, spontaneous bacterial peritonitis, and liver failure, eventually making the patients lose opportunities for other remedial measures. Thus, prediction of rebleeding and the identification of patients at high risk of rebleeding after endoscopic therapy are urgent issues that could help improve the prognosis of cirrhotic patients (3).

In clinical practice, the most important predictor for variceal rebleeding is the size of the varices determined with endoscopy (4). However, the compliance of patients is affected by its expensive and invasive properties. Hepatic venous pressure gradient (HVPG) has been widely proven to be a strong predictive factor for EV bleeding in patients with cirrhosis (5), but it is available only in specialized hepatology units, which restricts its widespread use (6). Some researchers explored several non-invasive models, such as the portal vein diameter (7), Child-Pugh score (8) and model for end-stage liver disease (MELD) score (9), to predict esophageal variceal rebleeding in cirrhotic patients. However, the predictive performance of these non-invasive tool is still controversial.

Radiomics is an emerging field that extracts innumerable quantitative medical features from imaging into high-dimensional data using many image characterization algorithms (10, 11). It has great diagnostic and prognostic value in many fields of non-neoplastic liver lesions, such as liver fibrosis (12, 13), hypertension and EV bleeding (14, 15). Most radiomics-related studies on EV bleeding have focused mainly on the prediction of the severity of EV and the presence of EV bleeding. However, the prediction of esophageal variceal rebleeding based on radiomics has not yet been reported.

In this study, we constructed and validated a radiomics score (Rad-score) derived from radiomics features of the liver and spleen in cirrhotic patients to predict the risk of rebleeding. Moreover, the Rad-score was used to stratify patients into high-, intermediate- and low-risk groups.

## Materials and Methods

This retrospective study was approved by the institutional review broad, and the requirement for written informed consent was waived.

### Patients

In this study, data from 173 patients diagnosed with cirrhosis between January 2011 and December 2019 were retrospectively analyzed. The patient inclusion criteria were as follows: (1) patients who had recovered from a first episode of EV bleeding and there was no bleeding for at least 5 consecutive days; (2) abdominal computed tomography (CT) scan and HVPG measurement were performed before endoscopic variceal ligation within 2 weeks; and (3) at least 1 year of follow-up after endoscopic therapies. The patient exclusion criteria were as follows: (1) previous therapy including splenectomy, endoscopic variceal ligation, tissue adhesive injection, or usage of non-selective beta blocker to prevent rebleeding; (2) confirmed to have hepatocellular carcinoma based on a histologic examination of the liver; (3) non-sinusoidal portal hypertension (e.g., hepatic cavernoma, Budd-Chiari syndrome); and (4) no contrast-enhanced CT images and HVPG measurement. The recruitment process is shown in Supplementary Figure 1.

### Definitions of Rebleeding and Therapy

The endpoint of the study was EV rebleeding during the 1-year follow-up. EV rebleeding is defined as the occurrence of new esophageal variceal bleeding after a period of 24 h or more from the 24-h point of stable vital signs and hematocrit/hemoglobin following the first episode of EV bleeding (4, 16).

The first episode of EV bleeding was controlled by measures including fluid resuscitation and medication administration (somatostatin and proton pump inhibitors). After recovering from a first episode of EV bleeding, all patients received secondary prevention of EV bleeding according to sixth Baveno Consensus (Baveno VI) (3), namely, the combination of endoscopic variceal ligation and non-selective beta blocker. Moreover, all hepatitis B-related cirrhotic patients received antiviral therapy.

Endoscopic examination was performed by experienced endoscopists. During the examination, the form, location and bleeding signs of varices were noted, and the size of the varices was classified as small, medium or large corresponding to <30, 30–60, or >60%, respectively, of the maximum theoretical size (17).

### CT Image Acquisition and Analysis

Contrast-enhanced CT scans were performed using a 320-detector CT scanner (Aquilion ONE, TOSHIBA) and a 64-detector CT scanner (Discovery, GE Healthcare). Non-enhanced CT scans were first acquired, followed by three post-contrast CT scans in three phases: arterial, portal vein and delayed. Arterial phase scanning started ~20–30 s after injection, portal phase scanning was started 30–40 s after the beginning of the arterial phase, and delayed phase scanning was started 40–60 s after the beginning of the portal phase scanning. The following parameters were used: tube voltage, 120 kV; tube current, 150–600 mAs; 80 × 0.5 mm or 64 × 0.625 mm detector collimation, matrix, 512 × 512; slice thickness, 5 mm; and pitch, 1.388 or 0.984. All patients received an intravenous, non-ionic contrast medium (iodine concentration, 370 mg/mL; volume, 1.5–2.0 mL/kg of body weight; Omnipaque 350, GE Healthcare, Shanghai, China) at a rate of 3–5 mL/s. Two imaging-based indexes including diameters of portal vein and spleen vein were assessed.

### Image Segmentation and Radiomics Feature Extraction

**Figure 1:**
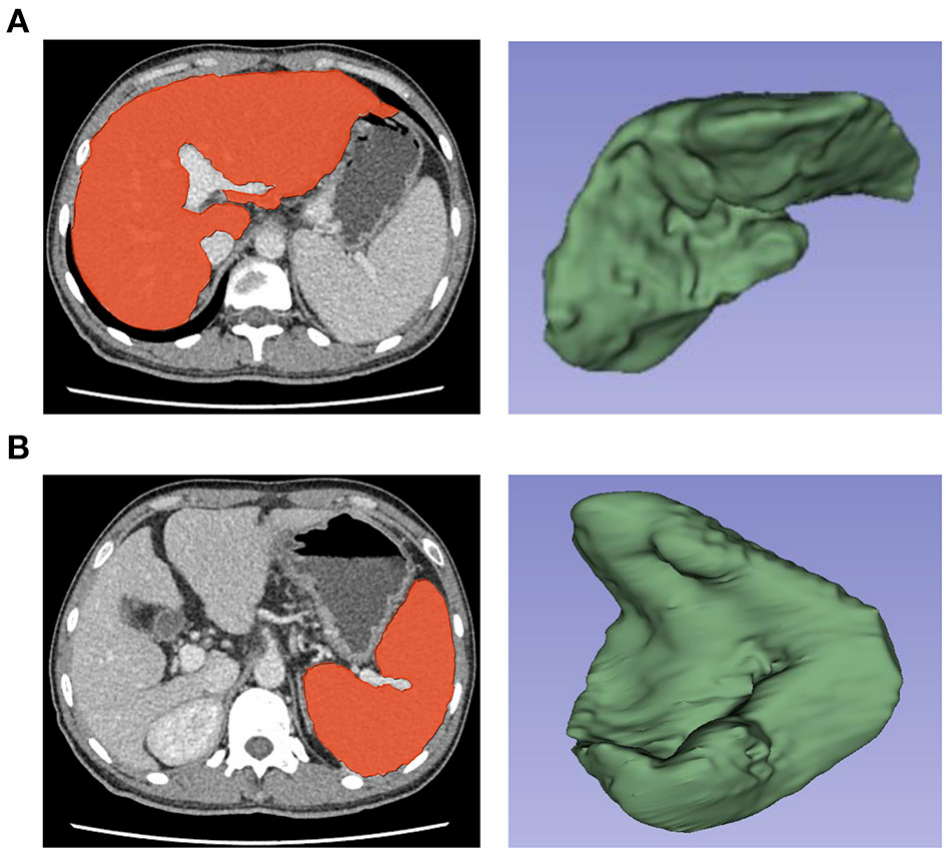
Selection and three-dimensional reconstruction of Regions of interest (ROIs) in the liver and spleen. Delineation of the liver **(A)** and spleen **(B)** as ROIs and three-dimensional reconstruction of ROIs by using 3D-slicer software for the extraction of radiomics features.

Regions of interest (ROIs) were drawn around the whole liver and spleen slice-by-slice using 3D-slicer software version 4.10.2 (Boston, USA) by a radiologist (Reader 1, Z.X.Y.) with 12 years of working experience in abdominal imaging. In ROIs of the liver and spleen, each ROI was as close as possible to the margin but excluded large vascular structures and artifacts to avoid adjacent organs such as the gallbladder, intestine, stomach, kidney and mesentery (Figure 1).

After image segmentation, we used Python 3.8.3 based on pyradiomics (version 3.0; https://pyradiomics.readthedocs.io/en/latest/index.html) for feature extraction. A total of 2,264 features were extracted from the liver and spleen ROIs (1,132 from each organ). Furthermore, the images of 173 patients were segmented by another radiologist (Reader 2, W.X.M.) who specialized in abdominal imaging and had 26 years of working experience to evaluate reproducibility. Reader 1 outlined the ROIs again after 1 month to minimize recall bias. The interobserver reproducibility and intraobserver reproducibility of all extracted features were evaluated by intra/interclass correlation coefficients (ICCs). Features with ICCs > 0.75 were considered to have good reproducibility. In the reproducibility analysis, a total of 1,882 features (953 from liver images and 929 features from spleen images) were found to be sufficiently reproducible and stable (ICCs > 0.75).

### Radiomics Feature Selection and Rad-Score Calculation

**Figure 2:**
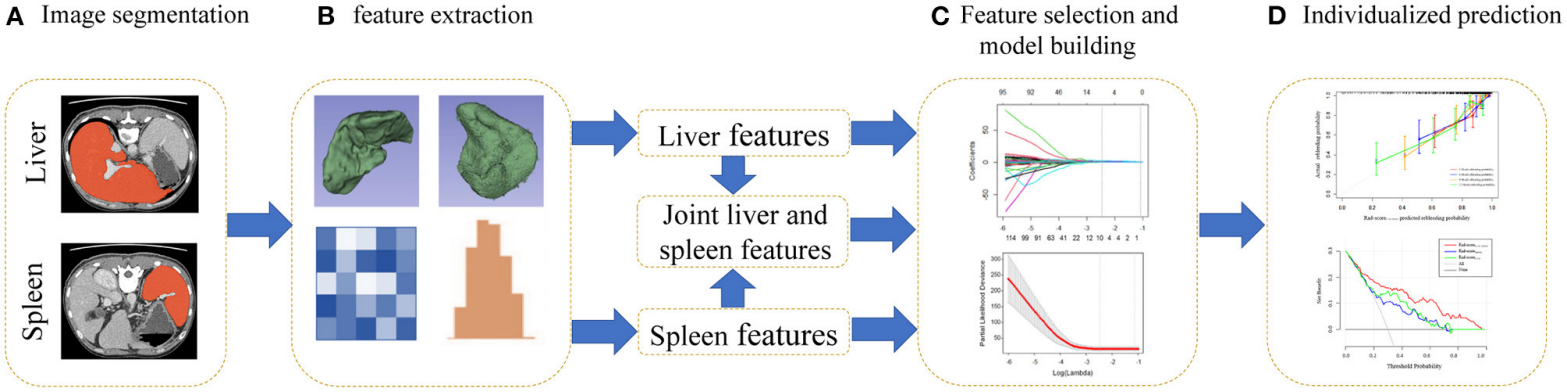
Flowchart for the radiomics analysis. **(A)** Regions of interest (ROIs) of the liver and spleen were segmented manually on all axial slices. **(B)** Three-dimensional reconstruction, texture analysis and feature extraction of ROIs. **(C)** For feature selection, the least absolute shrinkage and selection operator (LASSO) Cox method was used. A radiomics score was generated by a linear combination of selected features. **(D)** Calibration curves and decision curve analysis (DCA) were utilized to evaluate the Rad-score_Liver−Spleen_.

The radiomics workflow is presented in Figure 2. The training cohort was used for feature selection and model building, while the validation cohort was used to test model performance. To select the best features and avoid overfitting from the training cohort, we used the least absolute shrinkage and selection operator (LASSO) method and conducted 100 iterations of 10-fold cross-validations to develop a Lasso Cox regression model (18, 19). The coefficients of some features were decreased to zero by adding penalty terms through Lasso Cox regression, and the features with non-zero coefficients were then selected. Moreover, the optimal tuning parameter (λ) is the value for which the partial likelihood deviance is the minimum criterion. The significant features were weighted with their coefficients and summed to form the Rad-score (Rad-score = coefficient 1 × feature 1+ coefficient 2 × feature 2…) (20). The Rad-score_Liver_, Rad-score_Spleen_, and Rad-score_Liver−Spleen_ were calculated by a linear combination of the selected features from the liver, spleen and a combination of both organs that were weighted by their own coefficients in the LASSO Cox regression model.

### Assessment and Performance of the Rad-Score

Harrell's concordance index (C-index) and the hazard ratio (HR) were calculated to evaluate the predictive accuracy of the Rad-score. Kaplan–Meier survival analysis and the log-rank test were used to evaluate the stratification ability of each model. In addition, calibration curves were generated to assess the calibration of the Rad-score. Decision curve analysis (DCA) was performed to analyze the clinical usefulness of the Rad-score by measuring the net benefit at different threshold probabilities.

### Statistical Analysis

Statistical analysis was conducted with R software (version 3.6.1; http://www.r-project.org). The following R packages were used: glmnet, for running LASSO Cox; psych, for calculating ICCs; survival, for building the Cox proportional risk model and drawing Kaplan–Meier curves; hmisc, for calculating the C-index; rms, for generating calibration curves; stdca, for plotting DCA results; stats, for Mann–Whitney *U* and chi-square-tests; survcomp, for comparison of different C-indexes; and SurvProb, for predicting EV rebleeding probabilities. All statistical tests were two-sided, and *p*-values < 0.05 were considered significant.

## Results

### Study Patients

A total of 173 patients were divided into a training set and a validation set at a ratio of 7:3 with a random sampling method; 121 patients constituted the training cohort, and the other 52 constituted the validation cohort. There was no significant difference in clinical characteristics between the two cohorts (*p* = 0.212–0.868; Table 1). During the follow-up periods, rebleeding occurred in 39 of 173 patients (22.5%) within 1 year.

**Table 1:** Clinical characteristics of patients in the training and validation cohorts.

**Variable**	**Training** **(***n*** = 121)**	**Validation** **(***n*** = 52)**	***P*** **-value**
Mean age, years*	51.6 ± 11.6	50.4 ± 14.1	0.568
Sex (male/female)	83:38	35:17	0.868
Etiology, *n* (%)			0.243
Hepatitis B virus	72 (59.5)	32 (61.5)	
Alcoholism	23 (19.0)	5 (9.6)	
Hepatitis C virus	2 (1.7)	1 (1.9)	
Other	24 (19.8)	14 (27.0)	
Child-Pugh class, *n* (%)			0.827
A	63 (52.1)	29 (55.8)	
B	54 (44.6)	22 (42.3)	
C	4 (3.3)	1 (1.9)	
MELD score	8.7 ± 3.3	9.1 ± 3.1	0.165
AST (U/L)*	39.6 ± 40.5	43.2 ± 38.5	0.593
ALT (U/L)*	28.8 ± 21.8	31.0 ± 23.4	0.576
Creatinine (μmol/L)*	65.8 ± 16.9	64.6 ± 18.5	0.686
Hemoglobin (g/L)*	84.6 ± 23.7	84.2 ± 15.9	0.897
Platelet count (10^9^/L)*	77.8 ± 40.1	82.7 ± 58.0	0.522
EV size, *n* (%)			0.709
Small	1 (0.8)	0 (0)	
Medium	10 (8.3)	3 (5.8)	
Large	110 (90.9)	47 (94.2)	
HVPG (mmHg)	15.8 ± 0.4	15.5 ± 0.8	0.720
Portal vein diameter, mm*	14.8 ± 3.4	14.5 ± 3.9	0.527
Spleen vein diameter, mm*	9.6 ± 2.6	10.1 ± 2.3	0.212

* *Data are shown as the means ± standard deviations*.

*AST, aspartate aminotransferase; ALT, alanine aminotransferase; MELD, Model for End-Stage Liver Disease; EV, esophageal varices; HVPG, hepatic venous pressure gradient*.

### Radiomics Feature Extraction, Selection, and Rad-Score Calculation

After extracting features from ROIs, we obtained 7, 6, and 10 features with non-zero coefficients as the predictive radiomics features for the liver, spleen and both organs, respectively. The Rad-score formulas are follows:

1. Rad-score_Liver_ = −0.690 (Wavelet-LHH _Glrlm _RunEntropy)
−0.395 (original_glcm_JointAverage)
−0.250 (wavelet.HL_firstorder_Skewness)
+0.036(wavelet.LH_glcm_ClusterProminence)
+0.218 (log-sigma-4-0-mm-3D_glszm_LargeAreaEmphasis)
+0.535 (wavelet.HL_glszm_GrayLevelVariance)
+0.707 (wavelet-HHL_glcm_InverseVariance)
2. Rad-score_Spleen_ = −0.612 (log-sigma-2-0-Mm-3D_Firstorder_TotalEnergy)
−0.396 (wavelet-LHH_glszm_LargeAreaEmphasis)
+0.045 (wavelet-HLL _glszm_ZoneEntropy)
+0.162 (log-sigma-5-0-mm-3D_gldm_DependenceVariance)
+0.443 (wavelet-HL_firstorder_RootMeanSquared)
+0.540 (wavelet-HHL_glrlm_HighGrayLevelRunEmphasis)
3. Rad-score_Liver−Spleen_ = −0.529 (Wavelet-LHH_Glrlm_RunEntropy)
−0.318 (wavelet-LHH_glcm_JointEnergy)
−0.214 (log-sigma-2-0-mm-3D_firstorder_TotalEnergy)
−0.151 (wavelet.HL_firstorder_Skewness)
−0.093 (wavelet-LHH_glszm_LargeAreaEmphasis)
+0.102 (log-sigma-5-0-mm-3D_glrlm_RunVariance)
+0.139 (log-sigma-4-0-mm-3D_glszm_LargeAreaEmphasis)
+0.158 (wavelet-HHL_glrlm_HighGrayLevelRunEmphasis)
+0.432 (wavelet.HL_glszm_GrayLevelVariance)
+0.548 (wavelet-HHL_glcm_InverseVariance)

**Figure 3:**
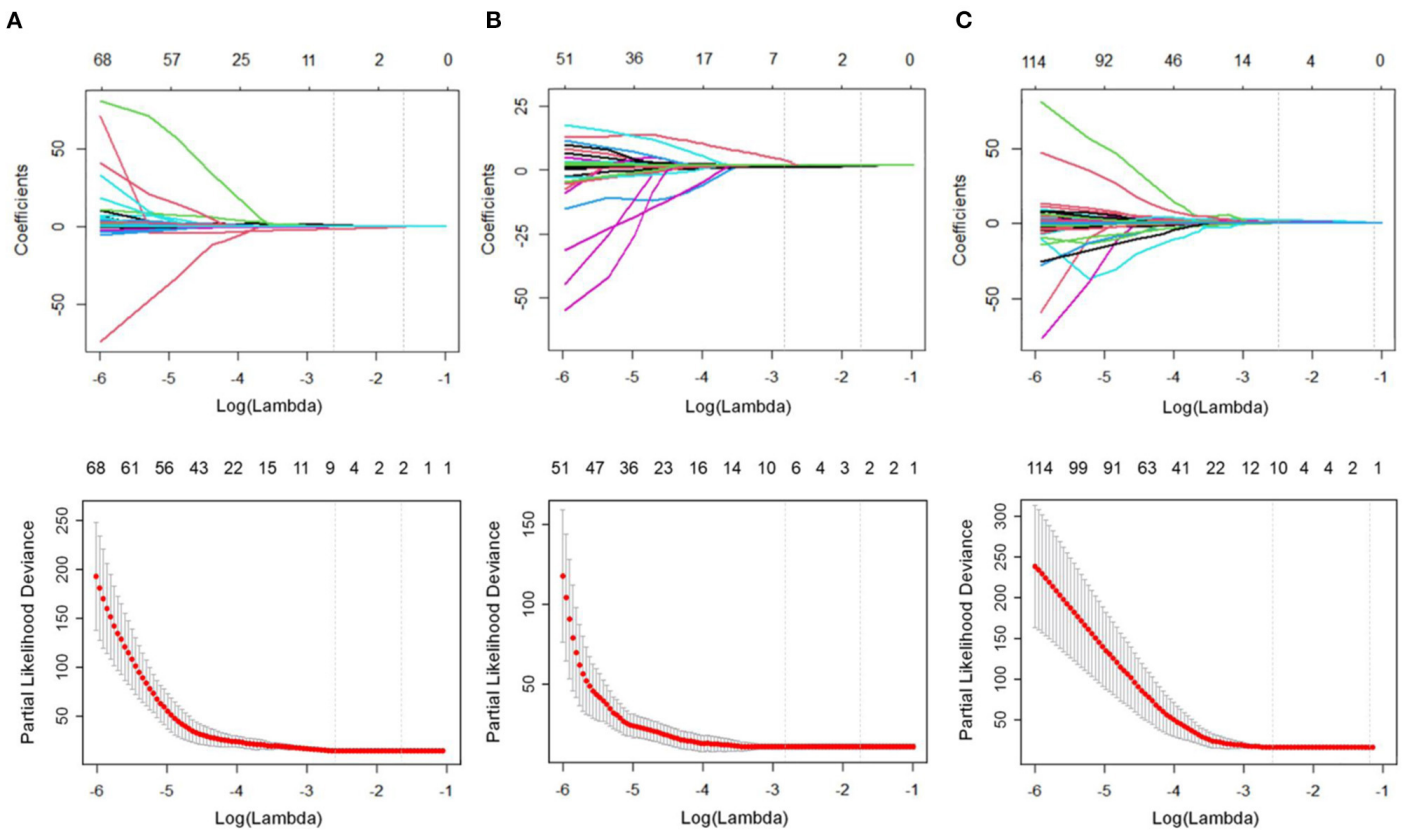
Feature selection using the least absolute shrinkage and selection operator (LASSO) Cox regression model. The partial likelihood deviance was plotted vs. log (λ). The tuning parameter (λ) was chosen in the LASSO Cox model *via* the minimum criteria. Dotted vertical lines were drawn at both the optimal and minimum values from left to right by using the minimum criteria and 1 standard error of the minimum criteria. **(A)** In the liver group, we examined the coefficients of the 953 radiomics features to identify 7 potential predictors. A λ-value of 0.0714, with log (λ), −2.6391, was chosen using 10-fold cross-validation. **(B)** In the spleen group, we examined the coefficients of the 929 radiomics features to identify 6 potential predictors. A λ-value of 0.0554, with log (λ) −2.8915, was chosen. **(C)** In the combined liver and spleen group, we examined the coefficients of the 1882 radiomics features to identify 10 potential predictors. A λ-value of 0.0675, with log (λ) −2.6956, was chosen.

Figure 3 represents the process of features selected with non-zero coefficients in the LASSO Cox regression model in the training cohort. The ICCs of the selected features are also described in Supplementary Table 1.

### Univariate and Multivariate Cox Regression Analysis of Rad-Score and Clinical Characteristics

Predictive factors for EV rebleeding are summarized in Table 2. In the univariate analysis, portal vein diameter [HR = 1.114; 95% confidence interval (CI) = 1.010–1.220; *p* = 0.002], Rad-score_Liver_ (HR = 1.692; 95% CI = 1.132–2.262; *p* < 0.001), Rad-score_Spleen_ (HR = 1.368; 95% CI = 1.019–1.741; *p* < 0.001) and Rad-score_Liver−Spleen_ (HR = 3.025; 95% CI = 2.029–3.961; *p* < 0.001) showed a significant association with EV rebleeding. In the multivariate analysis, Rad-score_Liver_ (HR = 1.355; 95% CI = 1.101–1.503; *p* =0.008), Rad-score_Spleen_ (HR = 1.148; 95% CI = 1.007–1.396; *p* =0.034) and Rad-score_Liver−Spleen_ (HR = 2.682; 95% CI = 1.793–3.512; *p* < 0.001) were independent risk factors for EV rebleeding.

**Table 2:** HR analysis of clinical characteristics and Rad-scores for predicting EV rebleeding.

**Variable**	**Univariate analysis**			**Multivariate analysis**		
	**HR**	**95%CI**	***P*-value**	**HR**	**95%CI**	***P-*value**
Age (years)	1.015	0.995–1.036	0.145			
Sex (male)	1.624	0.909–2.901	0.102			
Etiology						
Hepatitis B virus	1.008	0.537–1.894	0.980			
Alcoholism	1.292	0.595–2.802	0.517			
Hepatitis C virus	<0.001	0.001–27.230	0.965			
Other	Reference					
Child-Pugh class						
A	1.184	0.282–4.968	0.817			
B	0.897	0.214–3.761	0.882			
C	Reference					
MELD score	1.013	0.937–1.096	0.739			
AST (U/L)	1.002	0.995–1.008	0.629			
ALT (U/L)	0.998	0.990–1.006	0.615			
Creatinine (μmol/L)	1.008	0.994–1.022	0.260			
Hemoglobin (g/L)	1.001	0.988–1.011	0.962			
PLT (10^9^/L)	0.997	0.991–1.003	0.352			
EV size (large)	1.143	0.317–2.416	0.796			
HVPG (mmHg)	1.491	0.879–2.530	0.138			
Portal vein diameter (mm)	1.114	1.010–1.220	0.022*	1.017	0.805–1.229	0.327
Spleen vein diameter (mm)	0.970	0.902–1.044	0.416			
Rad-score_Liver_	1.692	1.132–2.262	<0.001*	1.355	1.101–1.503	0.008*
Rad-score_Spleen_	1.368	1.019–1.741	<0.001*	1.148	1.007–1.396	0.034*
Rad-score_Liver−Spleen_	3.025	2.029–3.961	<0.001*	2.682	1.793–3.512	<0.001*

* *Indicates p < 0.05*.

*AST, aspartate aminotransferase; ALT, alanine aminotransferase; MELD, Model for End-Stage Liver Disease; EV, esophageal varices; HVPG, hepatic venous pressure gradient; Rad-score, radiomics score*.

### Performance of the Rad-Score for EV Rebleeding

**Figure 4:**
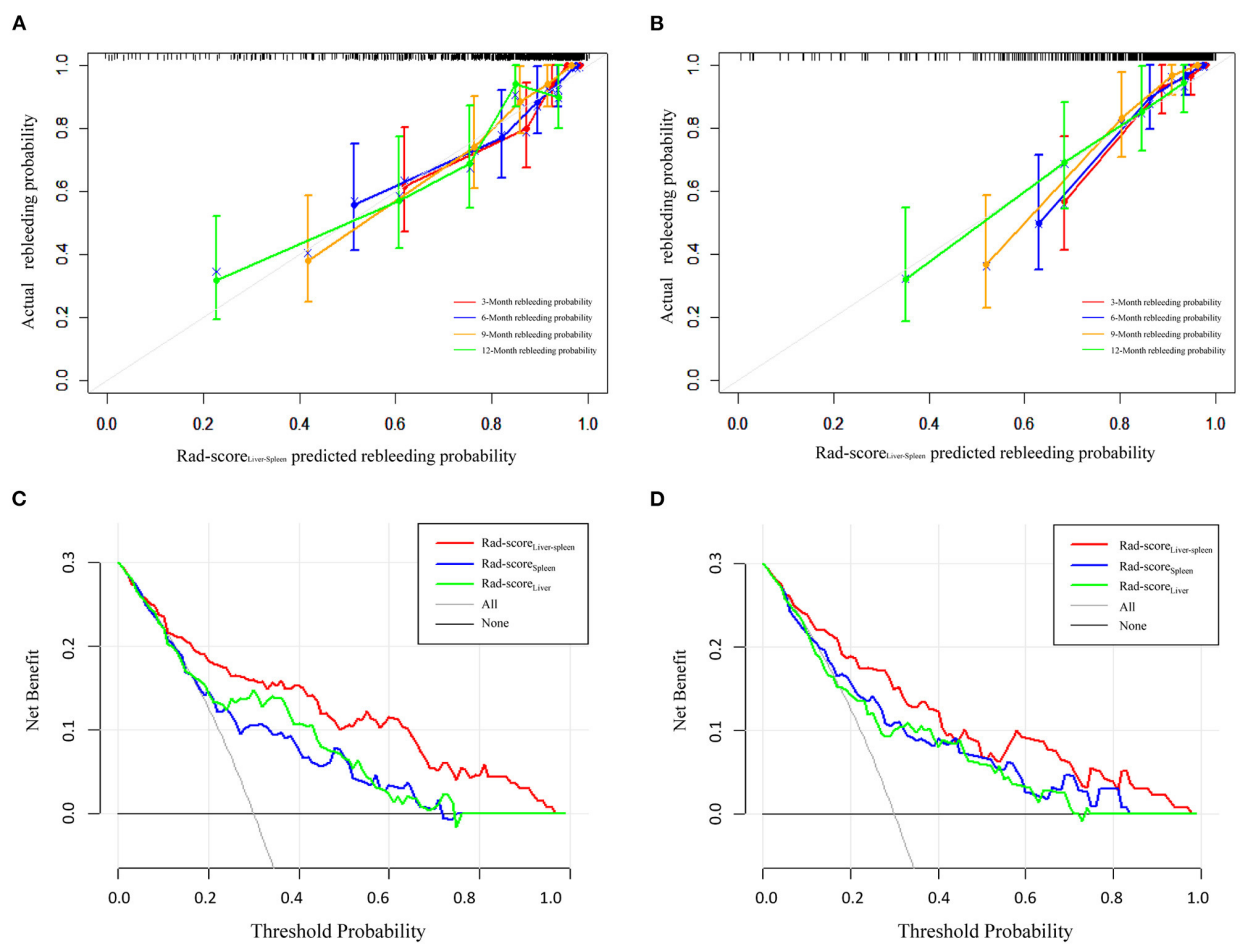
Calibration curves and decision curve analysis of the Rad-score_Liver−Spleen_. Calibration curves of the Rad-score_Liver−Spleen_ demonstrate its predictive performance for rebleeding at 3, 6, 9, and 12 months in the training cohort **(A)** and validation cohort **(B)**. Decision curve analysis was performed to compare the performance of the Rad-score_Liver−Spleen_, Rad-score_Liver_ and Rad-score_Spleen_ in the training cohort **(C)** and validation cohort **(D)**.

To compare the predictive performance of Rad-score_Liver_, Rad-score_Spleen_ and Rad-score_Liver−Spleen_ for EV rebleeding, the C-index was calculated. Rad-score_Liver−Spleen_ showed significantly better performance than Rad-score_Liver_ and Rad-score_Spleen_, yielding a C-index of 0.853 (95% CI = 0.776–0.904) in the training cohort and 0.822 (95% CI = 0.749–0.875) in the validation cohort (Table 3). The calibration curves of the Rad-score_Liver−Spleen_ at 3, 6, 9, and 12 months showed that the predicted probability was very close to the actual probability (Figures 4A,B). DCA showed that the Rad-score_Liver−Spleen_ yielded more clinical net benefit under almost all threshold probabilities, indicating that the Rad-score_Liver−Spleen_ is more practical than the Rad-score_Liver_ and Rad-score_Spleen_ for predicting esophageal variceal rebleeding in cirrhotic patients (Figures 4C,D).

**Table 3:** C-indexes of different models.

**Variable**	**Training cohort**			**Validation cohort**		
	**C-index**	**95% CI**	***P*** **-value**	**C-index**	**95% CI**	***P*** **-value**
HVPG model	0.575	0.467–0.684	<0.001*	0.545	0.431–0.659	<0.001*
Child-Pugh model	0.512	0.416–0.608	<0.001*	0.541	0.379–0.702	<0.001*
MELD model	0.520	0.406–0.633	<0.001*	0.515	0.403–0.627	<0.001*
EV size	0.544	0.528–0.560	<0.001*	0.533	0.503–0.6322	<0.001*
Rad-score_Liver_	0.784	0.708–0.855	0.021*	0.763	0.688–0.840	0.032*
Rad-score_Spleen_	0.766	0.684–0.848	0.018*	0.741	0.632–0.790	0.013*
Rad-score_Liver−Spleen_	0.853	0.776–0.904		0.822	0.749–0.875	

* *Indicates p < 0.05*.

*The p-value indicates the C-index of different models versus that of the Rad-score_Liver−Spleen_*.

*C-index, Harrell's concordance index; HVPG, hepatic venous pressure gradient; MELD, Model for End-Stage Liver Disease; EV, esophageal varices*.

We also used clinical indexes, including HVPG, Child-Pugh score, MELD score and EV size, to predict the probability of rebleeding. Compared with the clinical indexes, the Rad-score_Liver−Spleen_ exhibited a higher C-index in the training and validation cohorts (Table 3).

### Risk Stratification for Predicting EV Rebleeding According to Rad-Score_Liver-Spleen_

**Figure 5:**
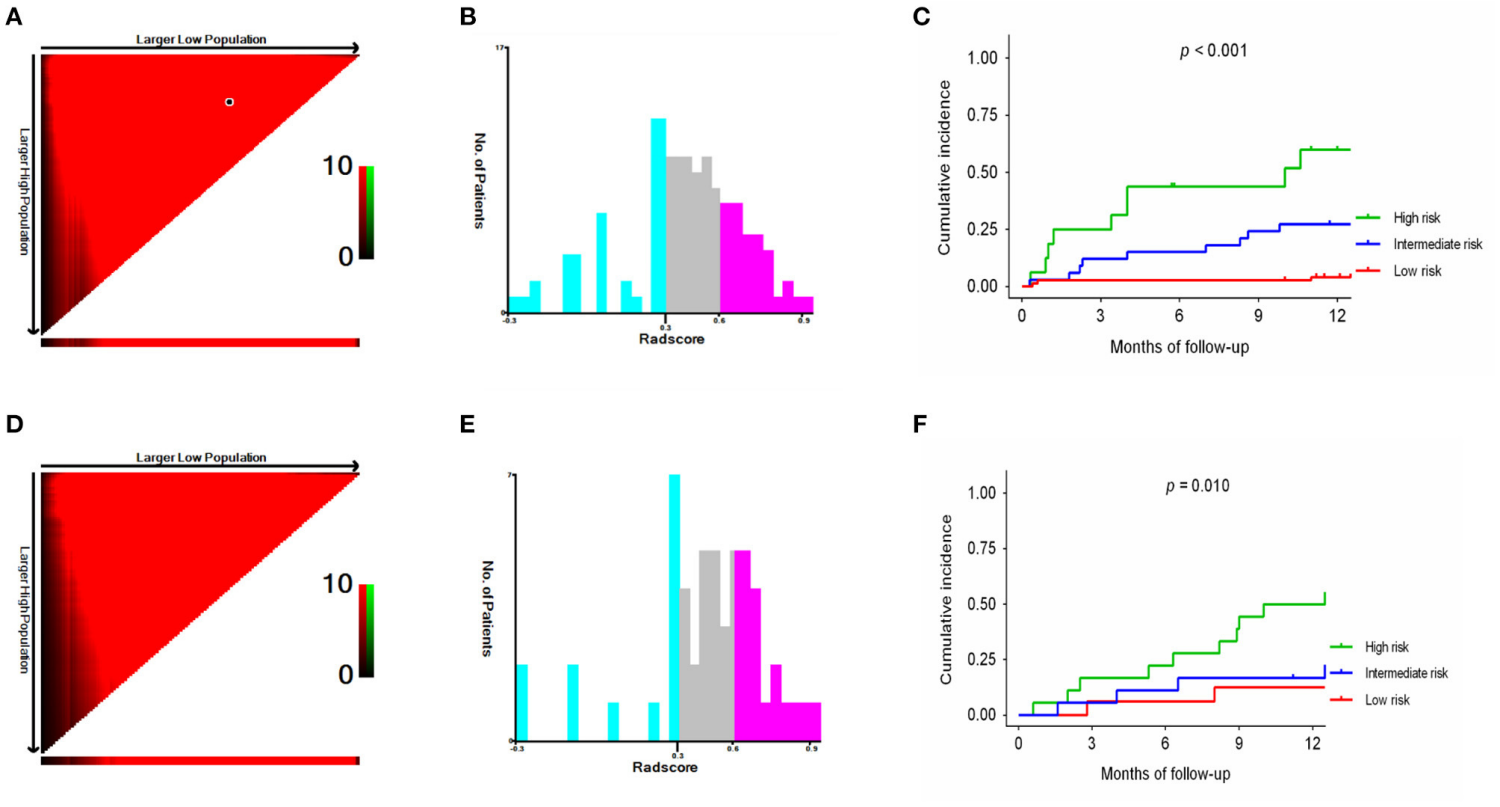
X-tile analysis of the total Rad-score_Liver−Spleen_ and survival curves stratified by the score calculated by the Rad-score_Liver−Spleen_ in the training **(A–C)** and validation **(D–F)** cohorts.

Based on the cutoff values of the Rad-score_Liver−Spleen_ determined by X-tile software (21), all patients were divided into 3 risk groups to predict rebleeding in the training (low-risk, −0.03–0.30; intermediate-risk, 0.31–0.61; high-risk, 0.62–0.9; Figures 5A,B) and validation (low-risk, −0.03–0.30; intermediate-risk, 0.31–0.61; high-risk, 0.62–0.9; Figures 5D,E) cohorts. The 12-month rebleeding probabilities among the 3 risk groups in the training cohort were 0.090, 0.202, and 0.407. Likewise, significant differences were observed in the validation cohort (12-month rebleeding probability: 0.097 for the low-risk group, 0.218 for the intermediate-risk group, and 0.436 for the high-risk group; Table 4). Kaplan–Meier curves showed that the cumulative incidences of rebleeding in the training and validation cohorts were accurately differentiated by the risk stratification system (Figures 5C,F).

**Table 4:** Rebleeding rates and probabilities according to the risk stratification.

	**No rebleeding,** *****n*** (%)**	**Rebleeding,** *****n*** (%)**	**3-month rebleeding** **probability (95% CI)**	**6-month rebleeding** **probability (95% CI)**	**9-month rebleeding** **probability (95% CI)**	**12-month rebleeding** **probability (95% CI)**
Training cohort						
Low risk	40 (83.3)	8 (16.7)	0.038 (0.034–0.042)	0.054 (0.048–0.060)	0.071 (0.064–0.079)	0.090 (0.080–0.010)
Intermediate risk	41 (71.9)	16 (28.1)	0.088 (0.083–0.093)	0.125 (0.094–0.132)	0.165 (0.153–0.172)	0.202 (0.191–0.213)
High risk	2 (12.5)	14 (87.5)	0.195 (0.166–0.223)	0.267 (0.230–0.303)	0.338 (0.295–0.380)	0.407 (0.359–0.454)
Validation cohort						
Low risk	16 (84.2)	3 (15.8)	0.041 (0.031–0.050)	0.066 (0.052–0.080)	0.081 (0.073–0.088)	0.097 (0.082–0.112)
Intermediate risk	17 (70.8)	7 (29.2)	0.093 (0.088–0.098)	0.134 (0.127–0.141)	0.178 (0.163–0.193)	0.218 (0.204–0.232)
High risk	1 (11.1)	8 (88.9)	0.189 (0.172–0.106)	0.286 (0.274–0.298)	0.349 (0.337–0.361)	0.436 (0.410–0.462)

### Subgroup Analysis for Predicting EV Rebleeding in Hepatitis B Virus and Non-HBV Cohorts

For the subgroup analysis in the training cohort, the Rad-score_Liver−Spleen_ in the HBV group had significantly better performance than that in the non-HBV group (C-index, 0.903 vs. C-index, 0.791; *P* < 0.001). Significant differences were also observed in the validation cohort (C-index, 0.884 vs. C-index, 0.781; *P* < 0.001, Table 5).

**Table 5:** Subgroup analysis of the C-indexes of the HBV and non-HBV cohorts.

**Variable**	**Training cohort**			**Validation cohort**		
	**HBV (95% CI)**	**Non-HBV (95% CI)**	***P-*value**	**HBV (95% CI)**	**Non-HBV (95% CI)**	***P*** **-value**
HVPG model	0.516 (0.458–0.574)	0.607 (0.540–0.674)	0.027*	0.508 (0.420–0.596)	0.561 (0.483–0.639)	0.423
Child-Pugh model	0.519 (0.456–0.582)	0.512 (0.450–0.574)	0.832	0.555 (0.484–0.626)	0.540 (0.472–0.608)	0.751
MELD model	0.637 (0.574–0.700)	0.597 (0.533–0.667)	0.482	0.575 (0.502–0.648)	0.503 (0.444–0.562)	0.271
EV size	0.568 (0.524–0.612)	0.512 (0.464–0.560)	0.039*	0.562 (0.488–0.632)	0.532 (0.480–0.584)	0.583
Rad-score_Liver_	0.832 (0.781–0.883)	0.769 (0.709–0.829)	0.018*	0.814 (0.756–0.872)	0.752 (0.689–0.815)	0.025*
Rad-score_Spleen_	0.818 (0.747–0.889)	0.728 (0.648–0.772)	0.033*	0.728 (0.648–0.772)	0.771 (0.719–0.823)	0.068
Rad-score_Liver−Spleen_	0.903 (0.870–0.937)	0.791 (0.732–0.850)	<0.001*	0.884 (0.821–0.947)	0.781 (0.723–0.839)	<0.001*

* *Indicates p < 0.05*.

*C-index, Harrell's concordance index; HVPG, hepatic venous pressure gradient; MELD, Model for End-Stage Liver Disease; EV, esophageal varices*.

## Discussion

Non-invasive tools for predicting EV rebleeding and risk stratification have been highlighted in recent years. The present study developed a Rad-score extracted from features of both the liver and spleen to predict EV rebleeding. Our results showed that Rad-score_Liver−Spleen_ was an independent significant predictive factor and achieved great predictive performance. In addition, Rad-score_Liver−Spleen_ could stratify patients into low-, intermediate- and high-risk groups for predicting rebleeding probability. Thus, the Rad-score_Liver−Spleen_ might be a promising tool to predict EV rebleeding in cirrhotic patients.

Based on the results of the LASSO Cox regression analysis, a total of 10 potential radiomics features were selected to calculate the Rad-score_Liver−Spleen_. Among these, run entropy, run variance and high gray level run emphasis measured the randomness and variance in the distribution of run lengths or higher gray-level values. Consistent with previous studies (14), a higher absolute value of high gray level run emphasis increased the possibility of EV bleeding. Joint energy and inverse variance were measures of homogeneous patterns in the image; if the image texture was relatively uniform and changed slowly between different regions, the inverse variance was increased. These features had a proper ratio for calculation of the Rad-score that could avoid overfitting and mainly reflected the texture complexity of the liver and spleen (15).

Our study revealed that Rad-score_Liver_, Rad-score_Spleen_, and Rad-score_Liver−Spleen_ were independent risk factors for EV rebleeding, suggesting that radiomics features of the liver and spleen were closely related to variceal bleeding. It was consistent with previous studies reporting that radiomics has a potential role in diagnosing portal hypertension and EV bleeding (14, 15, 22, 23). This finding could be explained by the hepatic-related factors and splenomegaly contributed to the rise of portal pressure in cirrhotic patients (24, 25). EV size and HVPG were not independent predictors in our study, which might be explained by the fact that non-selective beta blocker treatment can decrease portal blood flow and variceal pressure, leading to a change in hemodynamics.

Endoscopy and HVPG measurement which were reported to be predictors for EV rebleeding (4, 5, 26), are highly limited by their invasiveness and are therefore not suitable for dynamic monitoring. In contrast, Rad-score_Liver−Spleen_ is non-invasive and reproducible, it can extract quantitative features that reflect information related to all directions of the complex spatial structure of organs that are invisible to the human eye. Clinical physicians need only to upload CT images and select the ROI of the liver and spleen to perform the radiomics analysis and help to assess the risk of rebleeding in cirrhotic patients.

Cirrhotic patients usually undergo endoscopy every 3–6 months (16) after successful eradication of the varices. In order to reduce or avoid endoscopy examinations, it is of great significance for physicians to determine appropriate candidates for endoscopy according to risk stratification. In this study, Rad-score_Liver−Spleen_ divided all patients into low-, intermediate- and high-risk groups (3). Patients in the low-risk group could avoid endoscopy, while for patients in the high-risk group, endoscopy was performed as soon as possible to prevent rebleeding. For patients in the intermediate-risk group, regular follow-up should be carried out every 3–6 months until the Rad-score_Liver−Spleen_ reached the standard of the high-risk group.

In our study, 60.1% of patients had been infected with HBV, which remains the primary cause of cirrhosis in most Asian nations (27). Our results showed that Rad-score_Liver−Spleen_ had a significantly better performance in the HBV group than that in the non-HBV group, indicating that Rad-score_Liver−Spleen_ was particularly more suitable for the HBV population than for the non-HBV population.

There are several limitations to this study. First, this study was a single-center, retrospective analysis and subjected to the inherent limitations of such investigations. A multicenter, prospective study with a larger data set is needed. Second, we lacked hemodynamic data of the left gastric vein, portal vein, spleen vein, liver stiffness and spleen stiffness by ultrasound and transient elastography, which have proven to be good predictors of the degree of cirrhosis and the development of EV bleeding (28, 29). A future study comparing radiomics with other radiologic methods is needed.

## Conclusions

Our findings demonstrated that the Rad-score_Liver−Spleen_ could be used to predict the probability of EV rebleeding and stratify cirrhotic patients accordingly. The Rad-score_Liver−Spleen_ might serve as a useful tool for clinicians involved in therapeutic decision-making and individualized patient counseling.

## Data Availability Statement

The original contributions presented in the study are included in the article/Supplementary Material, further inquiries can be directed to the corresponding authors.

## Ethics Statement

The studies involving human participants were reviewed and approved by Biomedical Research Ethic Committee of Shandong Provincial Hospital. Written informed consent for participation was not required for this study in accordance with the national legislation and the institutional requirements. Written informed consent was not obtained from the individual(s) for the publication of any potentially identifiable images or data included in this article.

## Author Contributions

DM and XZ devised the experiment. DM and YW developed and organized the paper. XF and BK designed the tables and figures. XW and JQ performed the data analysis. XZ and QZ participated in the revision of the manuscript. DM and XZ wrote the original draft. All authors read and approved the final manuscript.

## Funding

This work was financially supported by the National Natural Science Foundation of China (81770607).

## Author Disclaimer

All claims expressed in this article are solely those of the authors and do not necessarily represent those of their affiliated organizations, or those of the publisher, the editors and the reviewers. Any product that may be evaluated in this article or claim that may be made by its manufacturer is not guaranteed or endorsed by the publisher.

## Conflict of Interest

The authors declare that the research was conducted in the absence of any commercial or financial relationships that could be construed as a potential conflict of interest.

## Publisher's Note

All claims expressed in this article are solely those of the authors and do not necessarily represent those of their affiliated organizations, or those of the publisher, the editors and the reviewers. Any product that may be evaluated in this article, or claim that may be made by its manufacturer, is not guaranteed or endorsed by the publisher.

## Supplementary Material

Supplementary Figure 1 Flowchart of the study population.

Supplementary Table 1 Intra/interclass correlation coefficients (ICCs) of the radiomics model.
