# Supplementary material for: CT-Based Radiomics Score Can Accurately Predict Esophageal Variceal Rebleeding in Cirrhotic Patients
Source: Front Med (Lausanne). 2021 Nov 4;8:745931. doi: 10.3389/fmed.2021.745931 (PMC8599938; doi:10.3389/fmed.2021.745931)
Supplement: Supplementary file 1 [file Presentation_1.pdf]

Supplementary Figure caption

Figure 1. Flowchart of the study population. HCC, hepatocellular carcinoma; CT, computed tomography (CT); NSBB, nonselective beta blocker; EV, esophageal varices.
