# Supplementary figures and images for: CT-Based Radiomics Score Can Accurately Predict Esophageal Variceal Rebleeding in Cirrhotic Patients
Source: Front Med (Lausanne). 2021 Nov 4;8:745931. doi: 10.3389/fmed.2021.745931 (PMC8599938; doi:10.3389/fmed.2021.745931)

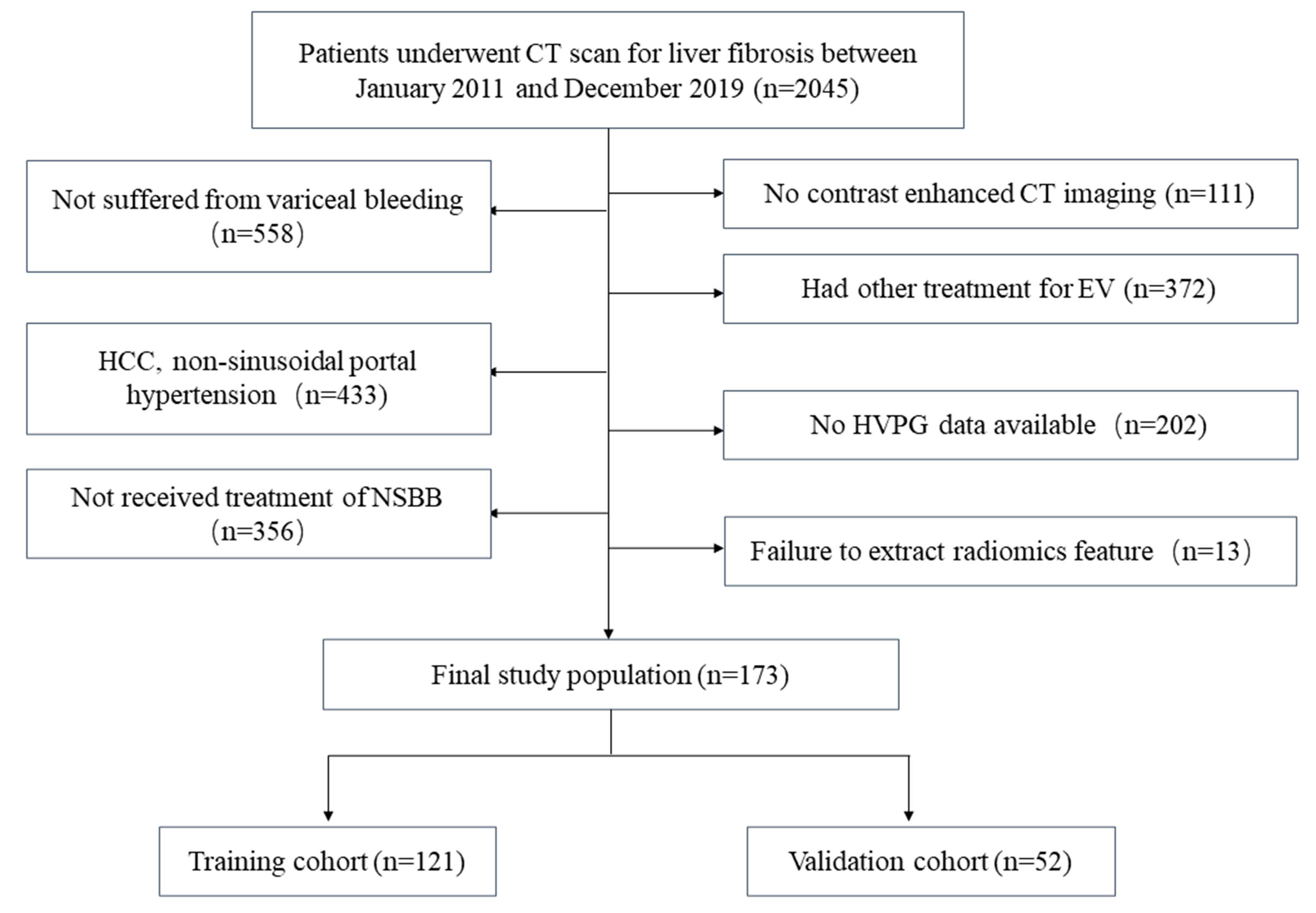

Supplement: Supplementary Figure 1 — Flowchart of the study population. [file Image_1.tif]
