# Supplementary material for: CT-Based Radiomics Score Can Accurately Predict Esophageal Variceal Rebleeding in Cirrhotic Patients
Source: Front Med (Lausanne). 2021 Nov 4;8:745931. doi: 10.3389/fmed.2021.745931 (PMC8599938; doi:10.3389/fmed.2021.745931)
Supplement: Supplementary Table 1 — Intra/interclass correlation coefficients (ICCs) of the radiomics model. [file Table_1.docx]

**Supplementary Materials**

**Table 1. Intra/interclass correlation coefficients (ICCs) of the radiomics model**

| Feature | Intraclass coefficient | Interclass coefficient |
| --- | --- | --- |
| Liver  wavelet-LHH_glrlm_RunEntropy | 0.8865145 | 0.8791288 |
| original_glcm_JointAverage | 0.9574069 | 0.8763519 |
| wavelet.HL_firstorder_Skewness | 0.9364236 | 0.9192402 |
| wavelet.LH_glcm_ClusterProminence | 0.8425384 | 0.8392341 |
| log-sigma-4-0-mm-3D_glszm_LargeAreaEmphasis | 0.8523417 | 0.8451591 |
| wavelet.HL_glszm_GrayLevelVariance | 0.9461972 | 0.8184236 |
| wavelet-HHL_glcm_InverseVariance | 0.8950219 | 0.9325013 |
| Spleen  log-sigma-2-0-mm-3D_firstorder_TotalEnergy  wavelet-LHH_glszm_LargeAreaEmphasis  wavelet-HLL_glszm_ZoneEntropy  log-sigma-5-0-mm-3D_gldm_DependenceVariance  wavelet-HL_firstorder_RootMeanSquared  wavelet-HHL_glrlm_HighGrayLevelRunEmphasis  Liver and spleen  wavelet-LHH_glrlm_RunEntropy  wavelet-LHH_glcm_JointEnergy  log-sigma-2-0-mm-3D_firstorder_TotalEnergy  wavelet.HL_firstorder_Skewness  wavelet-LHH_glszm_LargeAreaEmphasis  log-sigma-5-0-mm-3D_glrlm_RunVariance  log-sigma-4-0-mm-3D_glszm_LargeAreaEmphasis  wavelet-HHL_glrlm_HighGrayLevelRunEmphasis  wavelet.HL_glszm_GrayLevelVariance  wavelet-HHL_glcm_InverseVariance | 0.9595259  0.9135978  0.9321372  0.8851505  0.8586463  0.9259523  0.9058198  0.8473545  0.9546843  0.8944978  0.9828168  0.9566863  0.8575036  0.9172755  0.8599257  0.9698689 | 0.8352123  0.8476720  0.8308163  0.8531193  0.8567160  0.9011415  0.9452114  0.8295573  0.8917976  0.9584509  0.9162086  0.9471886  0.9359634  0.8932193  0.8720652  0.9289307 |

GLRLM, gray level run length matrix, GLCM; grayscale co-occurrence matrix, GLSZM, gray level size zone matrix; “LLH”, “HL” and “LH” represent the high-pass filter and low-pass filter on the three dimensions (X, Y, and Z). “H” represents the high-pass filter, and “L” represents the low-pass filter.
